# Assessment of bisphenol-related knowledge and awareness among healthcare professionals: a cross-sectional analysis from Türkiye

**DOI:** 10.3389/fpubh.2025.1627745

**Published:** 2025-09-12

**Authors:** Ekrem Aslan, Betül Kaplan

**Affiliations:** ^1^Graduate Education Institute, Hasan Kalyoncu University, Gaziantep, Türkiye; ^2^Department of Midwifery, Gaziantep Islamic Science and Technology University, Gaziantep, Türkiye; ^3^Department of Medical Services and Techniques, Vocational School, Hasan Kalyoncu University, Gaziantep, Türkiye

**Keywords:** bisphenols, healthcare workers, bisphenol knowledge level, healthcare professionals, environmental health

## Abstract

**Background:**

A class of endocrine-disrupting chemicals with numerous industrial uses and proven harmful health effects are bisphenols, especially bisphenol A (BPA). These substances pose serious exposure risks in healthcare settings because they are widely found in consumer goods, food containers, and medical equipment. Even though there is growing evidence that exposure to bisphenols can cause metabolic, reproductive, and cardiovascular problems, little is known about these environmental toxins by medical professionals. One major obstacle to efficient risk assessment, patient counseling, and occupational safety implementation in clinical settings is the lack of awareness among frontline healthcare workers.

**Methods:**

The healthcare professionals at Gaziantep University Şahinbey Research and Training Hospital participated in this descriptive cross-sectional study from April 15 to September 15, 2024. 397 healthcare professionals, including physicians, nurses, midwives, and health technicians, participated in standardized in-person interviews using a validated 13-item bisphenol knowledge assessment questionnaire. The data analysis was conducted using SPSS 25.0 software and included descriptive statistics, independent samples *t*-tests, one-way ANOVA, chi-square tests, Spearman correlation analysis, and CHAID decision tree analysis.

**Results:**

A critically low level of awareness was indicated by the fact that only 23.7% of participants reported having previously encountered bisphenols. With a mean knowledge score of 3.90 ± 3.48 out of 13 possible points, 82.6% of healthcare professionals were classified as having inadequate knowledge (*p* < 0.001). A significant difference in knowledge was observed based on professional title (physicians scored the highest, *p* = 0.015), marital status (married vs. single: 4.26 ± 3.57 vs. 3.39 ± 3.30, *p* = 0.014), and professional experience (6–10 years vs. 0–5 years: 4.29 ± 3.48 vs. 3.30 ± 3.15, *p* = 0.049). A positive correlation was observed between age and knowledge levels (*r* = 0.133, *p* = 0.008).

**Conclusion:**

The vast majority of Turkish healthcare professionals do not fully comprehend the sources of exposure, the health risks, or the precautions that should be taken about bisphenols, according to this study. These findings indicate that comprehensive educational interventions and policy reforms are urgently required to enhance environmental health literacy in healthcare settings.

## 1 Introduction

A variety of synthetic substances, referred to as “endocrine-disrupting chemicals” (EDCs), have the capacity to disrupt hormonal systems and have adverse effects on the immune system, development, reproduction, and nervous system (1). Bisphenol A (BPA) is one of the most widely researched and clinically significant environmental contaminants among these chemicals due to its established health effects and extensive commercial use (2, 3).

BPA is utilized in numerous industries, including the production of thermal receipt papers, epoxy resins, and polycarbonate plastics. Dental materials, laboratory equipment, medical devices, and intravenous tubing are frequently contaminated with BPA in healthcare environments. This presents a risk of occupational exposure for healthcare workers and iatrogenic exposure for patients (4, 5). The compound’s propensity to leach from plastic materials under a variety of circumstances, such as heating, aging, and contact with acidic or basic solutions, presents exposure risks in clinical settings where sterilization and chemical contact are routine (6).

The accumulation of epidemiological and experimental data has established a correlation between bisphenol exposure and a variety of adverse health consequences. Sirasanagandla et al. (2022) conducted a comprehensive review of the perinatal effects of BPA exposure on cardiovascular development. Their findings demonstrated that prenatal exposure can result in long-lasting cardiovascular programming changes in offspring, including altered blood pressure regulation, vascular function, and cardiac structure (7). These findings underscore the importance of healthcare professionals’ awareness, particularly pediatric and obstetric specialists who provide care to vulnerable populations.

Additionally, BPA exposure has been associated with metabolic diseases such as obesity, diabetes, and insulin resistance (8). Reproductive health effects encompass changes in pubertal timing, decreased fertility, pregnancy complications, and unfavorable birth outcomes (9, 10). The neurological effects include cognitive impairments, behavioral alterations, and neurodevelopmental disorders, while the immune system effects include heightened vulnerability to infections and autoimmune diseases (11, 12).

The introduction of bisphenol S (BPS) and bisphenol F (BPF), two BPA substitutes, has not entirely resolved health concerns due to their similar endocrine-disrupting qualities and the potential for equivalent or even greater health risks (8, 13). The evolving landscape of bisphenol compounds necessitates ongoing education and awareness among healthcare professionals.

International research has consistently documented substantial deficiencies in the knowledge of healthcare professionals regarding environmental health risks, such as bisphenols. Rouillon et al. conducted a comprehensive survey of French healthcare workers and found that, despite their critical role in patient risk assessment and counseling, 54.3% of them were unaware of endocrine-disrupting substances and 68.8% were unaware of their adverse health effects (14).

When Kumar et al. evaluated the knowledge, attitudes, and practices of dental surgeons in Southern India regarding BPA exposure, they discovered a lack of awareness in all areas studied. Insufficient comprehension of the sources of exposure and the health hazards was observed in even experts who were directly exposed to BPA-containing materials at work, according to their research (15). Marguillier et al. conducted a study in France that analyzed the knowledge of perinatal health professionals regarding endocrine disruptors. Their findings underscored the necessity of enhanced training to empower healthcare professionals to identify and mitigate environmental health risks for expectant mothers and their unborn children (16).

When Zhang et al. examined the knowledge of healthcare providers regarding environmental health risks in pediatric settings across multiple Asian countries, they consistently discovered that they were unaware of the impact of chemical exposures on development (17). Additionally, Brown et al. conducted an evaluation of environmental health literacy among Spanish nursing professionals, which revealed significant deficiencies in their comprehension of occupational hazards and patient exposure risks in healthcare environments (18).

There is a scarcity of research that has explicitly investigated the environmental health knowledge of healthcare professionals in Turkey, particularly in relation to bisphenols. When Turan Miral and Fışkın evaluated the awareness of Turkish healthcare workers regarding endocrine-disrupting chemicals, they discovered that their knowledge was generally inadequate. Nevertheless, their investigation did not concentrate on bisphenol compounds or their unique clinical implications; rather, it examined EDCs in general (19).

Rai et al. conducted an investigation into the occupational health awareness of Turkish hospital employees. Their findings revealed significant knowledge gaps regarding the hazards of chemical exposure in healthcare environments. Their research indicates that Turkish healthcare professional training programs do not adequately integrate environmental health education (20).

The Turkish healthcare system serves over 84 million individuals and comprises a network of public and private medical facilities. Healthcare providers in this system engage with diverse patient demographics, including vulnerable groups such as expectant mothers, newborns, and individuals with chronic illnesses who may be more prone to the adverse health impacts of bisphenols. Notwithstanding this obligation, the extent of Turkish healthcare professionals’ knowledge regarding bisphenols remains unclear.

This study addresses a substantial knowledge gap by providing the first comprehensive assessment of the awareness and knowledge of bisphenols among Turkish healthcare professionals. There are numerous reasons why the investigation is significant. Healthcare professionals are the primary sources of health information to patients and communities, and their comprehension of environmental health risks directly influences the quality of patient counseling, the accuracy of risk assessments, and the effectiveness of preventive care. It is imperative to comprehend these risks in order to implement the appropriate protective measures, as healthcare workers are also exposed to specific occupational risks as a result of their frequent contact with medical devices and equipment that contain BPA.

The level of environmental health literacy among healthcare professionals is a critical determinant of population health protection, as it impacts health outcomes and broader community awareness. In addition, the development of educational policies, the reformation of curricula, and the implementation of continuing education requirements can be facilitated by information regarding knowledge gaps among healthcare professionals. This study employs rigorous statistical analysis and validated assessment instruments to generate robust, broadly applicable findings that can inform the development of policies and interventions.

This research is innovative in that it is specifically focused on bisphenol knowledge in the Turkish healthcare context, thoroughly evaluates it across a variety of healthcare disciplines, and has the potential to inform the development of evidence-based educational interventions and health policies.

## 2 Methods

### 2.1 Study design and setting

This descriptive cross-sectional study was conducted at Gaziantep University Şahinbey Research and Training Hospital, a prominent tertiary care institution serving the southeastern region of Turkey. The hospital was selected as the study site due to its extensive clinical services, diverse medical workforce, and patient demographics that accurately represent the broader Turkish healthcare system.

### 2.2 Participants and sampling

Target Population: The study population comprised medical personnel at the research site, including physicians, nurses, midwives, and health technicians from various clinical departments.

Sample Size Calculation: Due to the absence of prior data regarding bisphenol awareness in this population, the sample size was determined using the formula for cross-sectional studies, incorporating a 95% confidence level, a 5% margin of error, and an assumed population proportion of 50%. The estimated minimum sample size was 325 participants.

Recruitment Strategy: Participants were selected from various hospital departments during regular business hours utilizing a convenience sampling method. The sample size was increased to 400 participants to mitigate potential non-response and ensure adequate representation across professional categories; ultimately, 397 participants completed the test.

#### 2.2.1 Inclusion criteria

Healthcare practitioners presently employed at the study location with a minimum of 6 months of experience.

Voluntary consent to participate.

Ability to communicate in Turkish.

#### 2.2.2 Exclusion criteria

Administrative staff not directly engaged in patient care.

Medical personnel on temporary assignments or extended leave refusing to provide informed consent.

### 2.3 Data collection instruments

#### 2.3.1 Sociodemographic characteristics questionnaire

The structural seven-item questionnaire was employed to collect the following demographic and professional data: years of professional experience (0–5, 6–10, 11–15, 16 + years), income level (low, medium, high), gender (male/female), marital status (married/single), professional title (physician, nurse, midwife, health technician), educational level (high school, associate degree, bachelor’s degree, postgraduate), and prior bisphenol awareness (yes/no/do not know).

#### 2.3.2 Bisphenol knowledge assessment questionnaire

The development of the 13-item knowledge assessment questionnaire involved a comprehensive review of existing bisphenol knowledge assessment tools, expert consultations with environmental health specialists, content validation by three public health academics, and pilot testing with 20 healthcare professionals (excluded from the final analysis).

The questionnaire assessed three primary domains of knowledge. The initial domain evaluated knowledge regarding BPA-containing products and materials commonly present in healthcare and daily settings, focussing on exposure sources. The second domain, health effects, assessed the understanding of the known and potential health impacts of bisphenol exposure on various organ systems and throughout different life stages. The third domain, vulnerable populations, examined awareness of groups at heightened risk for bisphenol-related health effects, including pregnant women, infants, and individuals with specific medical conditions.

The questionnaire employed a binary scoring system, assigning 1 point for correct responses (“Yes” when applicable) and 0 points for incorrect or ambiguous responses (“No” or “I do not know”), consistent with established methodologies in environmental health knowledge assessment (21–24). A total score of 13 points was attainable, with a knowledge adequacy threshold set at ≥8 points (≥60% correct responses).

Three scholars with expertise in public health education, occupational medicine, and environmental health performed an expert review to assess content validity. Experts evaluated each item for its difficulty level, clarity and comprehensibility for healthcare professionals, absence of cultural or linguistic bias, and relevance to bisphenol exposure and health effects.

This study did not encompass formal psychometric validation, such as factor analysis, test–retest reliability, and internal consistency evaluation, despite addressing content and face validity through expert consultation. This limitation is acknowledged and constitutes a subject for additional investigation.

### 2.4 Data collection procedures

#### 2.4.1 Interview protocol development

A detailed interview protocol ensured data collection consistency. The protocol included scripted instructions for administering the questionnaire, uniform response recording procedures, a standardized introduction explaining the study’s purpose and confidentiality, and guidelines for participant questions and clarifications.

To maintain confidentiality, each interview was conducted in a private room with consent forms, questionnaires, and writing materials, the interviewer reviewed the protocol, and 15–20 min per participant was allotted to avoid rushing.

#### 2.4.2 Interview implementation

Each in-person interview lasted 10 to 15 min in a private office or conference room to ensure confidentiality and minimize distractions.

The interviewer sequentially read each questionnaire question aloud and documented the responses verbatim. We eschewed prompts, visual stimuli, and suggestive language to mitigate response bias.

The principal researcher (EA) conducted all interviews. Interviewers were instructed to pose neutral inquiries, refrain from non-verbal signals that might sway responses, sustain a uniform tone and pace during interviews, and address participant questions suitably without disclosing pertinent study information.

All participant responses were documented on standardized forms featuring pre-coded options. Audio interviews were not documented to safeguard participant privacy and minimize response inhibition.

#### 2.4.3 Timing and scheduling

Data collection was conducted from April 15 to September 15, 2024, which provided all hospital departments and professional categories with an opportunity to recruit.

In order to optimize participation and mitigate clinical interference, interviews were conducted during standard business hours. Shift patterns and departmental requirements were accommodated by flexible scheduling.

### 2.5 Bias control and quality assurance

Various approaches were employed to mitigate bias and uphold methodological rigor. Participants were recruited from various major hospital departments to ensure a diverse clinical and professional composition. Due to the absence of rewards or penalties, participation was entirely voluntary, thereby minimizing external selection bias.

The principal investigator received training in neutral interviewing techniques, which encompassed standardizing participant questions and clarifications, refraining from verbal or nonverbal cues that could suggest preferred responses, and sustaining a consistent tone and tempo throughout the process. The absence of “right” or “wrong” answers, assurance of anonymity and confidentiality, clarification that responses would not influence professional evaluations, and the conduct of interviews in private collectively mitigated social desirability bias. The questionnaire was designed as a knowledge assessment instead of a competency test to mitigate anxiety and response distortion.

To mitigate the confounding effects of varying data collection methods, all interviews adhered to uniform protocols, question presentation, and environmental conditions. The data collection process was finalized within 5 months to minimize external influences and the impact of evolving knowledge on the results.

### 2.6 Statistical analysis plan

The research data were analyzed utilizing the Statistical Package for Social Sciences (SPSS) version 25.0. Continuous variables were summarized using the mean (x̄) and standard deviation (SD), while categorical variables were summarized with frequency (n) and percentage (%) values. An Independent Samples t-Test was employed to compare two independent groups (e.g., gender: female and male). One-Way Analysis of Variance (One-Way ANOVA) was utilized for variables including three or more groups (e.g., income level: low, medium, high). In instances where normal distribution was present but variances were not homogeneously distributed, the Welch ANOVA test was utilized (17–19). *Post-hoc* multiple comparison tests, including Bonferroni and Games-Howell, were employed to identify the cause of differences across the groups; disparities were denoted by the numerals “1-2-3-4” and the “>” (greater than) sign. The Chi-square test was employed to investigate the association between categorical variables, while Spearman correlation analysis was utilized to assess the link between ordinal or non-normally distributed data. Furthermore, CHAID (Chi-squared Automatic Interaction Detection) decision tree analysis was used to identify the factors influencing healthcare professionals’ understanding of bisphenol. In all statistical analyses, the significance threshold was established at *p* < 0.05.

## 3 Results

### 3.1 Participant characteristics and demographics

A total of 397 medical professionals from diverse clinical specialties and varying levels of experience participated in this cross-sectional survey. The participants had a mean age of 31.34 ± 6.02 years, with an age range of 21 to 56 years, indicating a relatively young professional workforce consistent with the recent expansion of Turkey’s healthcare system. The sample consisted of 220 female participants (55.4%) and 177 male participants (44.6%), reflecting the typical gender distribution observed in Turkish healthcare facilities, where women are predominantly found in medical and technical roles, as well as in nursing and midwifery.

The educational backgrounds of participants varied considerably: 32 (8.1%) had completed only high school, 94 (23.7%) held associate degrees, 216 (54.4%) possessed bachelor’s degrees, and 55 (13.9%) had attended graduate school. This distribution reflects the diverse educational pathways present in Turkish healthcare professions. The sample comprised 126 doctors (31.7%), 116 nurses (20.2%), 30 midwives (7.6%), and 125 health technicians (31.5%). The relatively low number of midwives in the Turkish healthcare system reflects the specialized nature of the profession.

The distribution of experience levels is outlined below: Among the participants, 169 (42.6%) had 0–5 years of experience, 139 (35.0%) had 6–10 years, 51 (12.8%) had 11–15 years, and 38 (9.6%) had 16 years or more. The distribution indicates a strong representation of both experienced and early-career professionals within the workforce. To ensure balanced representation across marital status categories for comparative analysis, 232 participants (58.4%) were married, while 165 participants (41.6%) were single. A total of 157 participants (39.5%) reported low incomes, 221 participants (55.7%) reported medium incomes, and 19 participants (4.8%) reported high incomes. The prevalence of low-to-medium income groups reflects prevailing compensation trends within the Turkish public health care system.

A total of 94 participants (23.7%) reported prior knowledge of bisphenol chemicals, while 233 participants (58.7%) indicated they were unaware, and 70 participants (17.6%) stated they had no knowledge of them. This baseline result highlighted substantial knowledge gaps requiring thorough investigation. The detailed distribution of these findings is presented in Table 1.

**Table 1:** Sociodemographic characteristics of healthcare workers (*n* = 397).

Characteristic	Category	n	%
Age(years)	Mean ± SD	31.34 ± 6.02	_
	Range	21–56	**_**
Gender	Female	220	55.4
	Male	177	44.6
Marital Status	Married	232	58.4
	Single	165	41.6
Title	Doctor	126	31.7
	Nurse	116	29.2
	Midwife	30	7.6
	Health Technicians	125	31.5
Professional Experience	0–5 years	169	42.6
	6–10 years	139	35.0
	11–15 years	51	12.8
	16 years and above	38	9.6
Have you heard of a chemical called Bisphenol before?	Yes	94	23.7
	No	233	58.7
	I do not know	70	17.6

SD, Standard deviation; n, number of participants; %, percentage.

### 3.2 Bisphenol knowledge assessment results

The 13-item Bisphenol Knowledge Assessment revealed significant knowledge gaps among the research participants. The mean score for each item was 0.30 ± 0.27 (range 0–1), indicating that, on average, respondents answered less than one-third of the knowledge evaluation items correctly. The mean score for knowledge was 3.90 ± 3.48 out of a maximum of 13 points (range: 0–12), with a median score of 3.0 points (IQR: 1–7). The central tendency analysis indicates that 50% of test participants scored 3 points or fewer, reflecting an accuracy rate of less than 25%.

An analysis of the score distribution revealed that 89 participants (22.4%) scored between 0 and 1 points, 127 participants (32.0%) scored between 2 and 4 points, 112 participants (28.2%) scored between 5 and 7 points, and only 69 participants (17.4%) scored 8 or higher. The distribution indicates that the entire study population exhibited a deficiency in information, rather than specific subgroups alone.

According to the established criterion of ≥8 correct answers (≥60% accuracy) for determining adequate knowledge, 328 individuals (82.6%) demonstrated insufficient knowledge regarding bisphenol, whereas only 69 participants (17.4%) exhibited sufficient knowledge levels. The result was, indicating a substantial lack of knowledge among healthcare professionals regarding this matter. The 82.6% rate of insufficient knowledge is a significant finding that impacts patient safety, workplace health protection, and the healthcare system’s capacity to educate the public about health. The detailed distribution of these findings is presented in Table 2.

**Table 2:** Distribution of bisphenol knowledge scores among healthcare professionals (*n* = 397).

Item	No. of items	Mean ± SD	Range	Total Score (Mean ± SD)	Min–Max	Median [IQR]
BPA Knowledge Scale	13	0.30 ± 0.27	0–1	3.90 ± 3.48	0–12	3 [7–1]
Knowledge level					Frequency (n)	(%)
Inadequate					328	82.6
Adequate					69	17.4

Mean: Average; SD, Standard Deviation; Min, Minimum; Max, Maximum; M (IQR), Median (Interquartile Range, 75th and 25th percentiles).

### 3.3 Knowledge differences by sociodemographic characteristics

The mean knowledge score for women was 3.82 ± 3.54, while for men it was 4.01 ± 3.42. The independent samples t-test indicated no statistically significant difference between men and women (*t* = −0.533, *p* = 0.594; Table 3). This indicates that male and female healthcare workers exhibited equivalent knowledge gaps. The proportions of acceptable knowledge were comparable for both genders. 18.2% of women and 16.4% of men achieved scores that met the criteria (χ^2^ = 0.113, *p* = 0.736; Table 4).

**Table 3:** Distribution of bisphenol knowledge scores by sociodemographic characteristics of healthcare workers (*n* = 397).

Categorical variables	n	Mean ± SD	Statistical test	Difference
Gender				
Female	220	3.82 ± 3.54	*t* = −0.533 *p* = 0.594	-
Male	177	4.01 ± 3.42		
Marital status				
Married	232	4.26 ± 3.57	*t* = 2.467 *p* = 0.014	-
Single	165	3.39 ± 3.30		
Title				
Physician^1^	126	4.64 ± 3.61	F = 3.231 *p* = 0.022	1 > 2; *p* = 0.015
Nurse^2^	116	3.29 ± 3.54		
Midwife^3^	30	3.77 ± 3.19		
Health Technician^4^	125	3.75 ± 3.26		
Years of professional experience				
0–5 years^1^	169	3.30 ± 3.15	W = 3.116	2 > 1; *p* = 0.049
6–10 years^2^	139	4.29 ± 3.48	*p* = 0.029	
11–15 years^3^	51	4.33 ± 3.53		
16 years and above^4^	38	4.61 ± 4.41		
Continuous Variable			Bisphenol knowledge level form	
			Correlation (r)	*p*-value
Age			0.133**	0.008

Statistically significant correlation at the *p* < 0.01 threshold. Mean, SD, Standard Deviation; n, Sample Size; t, Independent Samples *t*-test statistic; F, One-way analysis of variance (ANOVA) value; W, Welch test value 1-2-3-4: Indication of intergroup differences. **Spearman correlation analysis. *Post-hoc*: Intergroup comparison (Bonferroni and Games-Howell methods).

**Table 4:** Association between socio-demographic characteristics and bisphenol knowledge adequacy among healthcare professionals.

Variable	Bisphenol knowledge level						
	Inadequate knowledge		Adequate knowledge		Total		
	n	%	n	%	n	%	Test result
Gender							
Female	180	81.8	40	18.2	220	100.0	χ^2^ = 0.113
Male	148	83.6	29	16.4	177	100.0	*p* = 0.736
Marital Status							
Married	184	79.3	48	20.7	232	100.0	χ^2^ = 3.721
Single	144	87.3	21	12.7	165	100.0	*p* = 0.054
Title							
Doctor	99	78.6	27	21.4	126	100.0	χ^2^ = 2.694
Nurse	96	82.8	20	17.2	116	100.0	*p* = 0.441
Midwife	25	83.3	5	16.7	30	100.0	
Health Technicians	108	86.4	17	13.6	125	100.0	
Professional experience							
0–5 years	150	88.8	19	11.2	169	100.0	χ^2^ = 9.325
6–10 years	110	79.1	29	20.9	139	100.0	*p* = 0.025
11–15 years	41	80.4	10	19.6	51	100.0	
16 years and above	27	71.1	11	28.9	38	100.0	

n, Number; %, Percentage; χ^2^, Chi-square test; ^1^Pearson chi-square test value (for 3 × 2 tables), ^2^Continuity correction test value (for 2 × 2 tables).

Married individuals demonstrated significantly higher scores on knowledge assessments compared to their single counterparts (4.26 ± 3.57 vs. 3.39 ± 3.30, *t* = 2.467, *p* = 0.014; Table 3). The 0.87-point difference represents a statistically significant and potentially clinically relevant enhancement in knowledge performance. The proportion of individuals possessing sufficient knowledge varied according to marital status. For instance, 20.7% of married individuals possessed sufficient knowledge, while only 12.7% of unmarried individuals did. The observed difference approached statistical significance (χ^2^ = 3.721, *p* = 0.054; Table 4). The relationship between marital status and knowledge levels may be attributed to several factors, including increased life experience, heightened health consciousness due to familial responsibilities, or greater access to health-related information via spousal networks.

Significant differences in knowledge ratings were observed among various professional categories (*F* = 3.231, *p* = 0.022). The average score for doctors was the highest at 4.64 ± 3.61, followed by health technicians at 3.75 ± 3.26, midwives at 3.77 ± 3.19, and nurses at 3.29 ± 3.54 Bonferroni *post hoc* testing indicated that physicians had significantly higher scores than nurses (mean difference = 1.35, *p* = 0.015; Table 3). No statistically significant differences were observed in other pairwise comparisons. The knowledge adequacy rates varied among professions: 21.4% for doctors, 16.7% for midwives, 13.6% for health technicians, and 17.2% for nurses. However, these differences were not statistically significant (χ^2^ = 2.694, *p* = 0.441; Table 4). Doctors may outperform other healthcare professionals due to their extensive training in pathophysiology and toxicology. This indicates that educational interventions must be customized to align with the distinct educational backgrounds and clinical duties of various healthcare professional groups.

Significant disparities in knowledge ratings were observed among groups with varying levels of experience (Welch *F* = 3.116, *p* = 0.029). Healthcare workers with 6–10 years of experience demonstrated significantly higher scores compared to those with 0–5 years of experience (4.29 ± 3.48 vs. 3.30 ± 3.15, *p* = 0.049 in *post hoc* analysis). The mean knowledge scores corresponding to various experience levels were as follows: 0–5 years (3.30 ± 3.15), 6–10 years (4.29 ± 3.48), 11–15 years (4.33 ± 3.53), and 16 + years (4.61 ± 4.41; Table 3). The data indicates that individuals acquire the majority of their knowledge during the early to mid-stages of their careers, after which their learning tends to decline significantly. As professionals accumulated experience, their knowledge adequacy rates increased: 0–5 years (11.2%), 6–10 years (20.9%), 11–15 years (19.6%), and 16 years or more (28.9%). The observed change was statistically significant (χ^2^ = 9.325, *p* = 0.025), indicating that engagement in a professional environment enhances knowledge of environmental health (Table 4).

Spearman correlation analysis revealed a statistically significant positive correlation between age and bisphenol knowledge scores (rho = 0.133, *p* = 0.008; Table 3). This association is considered weak; however, it indicates that knowledge levels generally increase with age. This may be attributed to the accumulation of life experiences and professional development over time.

### 3.4 Knowledge adequacy and demographic associations

The duration of professional experience emerged as the most significant demographic factor influencing knowledge adequacy. Chi-square analysis indicated significant associations between experience levels and knowledge adequacy (χ^2^ = 9.325, *p* = 0.025). The lowest adequacy percentage recorded was 11.2% (19 out of 169) for healthcare workers with 0–5 years of experience. The highest rate, 28.9% (11 of 38), was observed among individuals with 16 or more years of experience. The middle groups included individuals with 6–10 years (29 out of 139, 20.9%) and 11–15 years (10 out of 51, 19.6%; Table 4). The findings indicate that current basic professional education programs inadequately prepare healthcare personnel for environmental health challenges. They primarily acquire knowledge through professional experience and ongoing educational opportunities.

The association between marital status and knowledge adequacy approaches statistical significance (χ^2^ = 3.721, *p* = 0.054). Married individuals demonstrated a higher likelihood of being adequate (48 out of 232, or 20.7%) compared to single individuals (21 out of 165, or 12.7%; Table 4). The trend can be attributed to several factors: individuals may exhibit increased health awareness due to family responsibilities, receive health information through spouses and family networks, seek knowledge to safeguard their families, or simply reflect the demographic tendency of married individuals being older.

### 3.5 Correlation analysis and relationship patterns

Spearman correlation analysis indicated that age (rho = 0.113, *p* = 0.025), professional experience (rho = 0.144, *p* = 0.004), and marital status (rho = −0.104, *p* = 0.039, with single status receiving higher scores) significantly influenced bisphenol knowledge levels. Significant correlations existed across demographic characteristics: age and professional experience (rho = 0.774, *p* < 0.001), age and marriage status (rho = −0.566, *p* < 0.001), and professional experience and marital status (rho = −0.507, *p* < 0.001). The correlations indicate that the knowledge acquired in regard to each variable may stem from factors such as maturation or life events, rather than being directly attributable to the variables themselves. A little negative correlation existed between professional title and knowledge scores (rho = −0.082, *p* = 0.104), although this relationship was not statistically significant. The detailed distribution of these findings is presented in Table 5.

**Table 5:** The relationship between sociodemographic variables of healthcare workers and information level.

Variables		Information level		Marital status		Professional experience
Information level	rho	1				
	*p*					
Age	rho	0.113^*^	1			
	*p*	0.025				
Marital status	rho	−0.104^*^	−0.566^**^	1		
	*p*	0.039	<0.001			
Title	rho	−0.082	−0.075	0.049	1	
	*p*	0.104	0.136	0.327		
Professional experience	rho	0.144^**^	0.774^**^	−0.507^**^	−0.020	1
	*p*	0.004	<0.001	<0.001	0.685	

*p < 0.05 and **p < 0.01 indicate statistically significant relationships. Interpretation of ‘r’: 0.10 ≤ r < 0.30 (weak, small), 0.30 ≤ r < 0.50 (moderate), 0.50 ≤ r (large, strong) relationship.

### 3.6 Decision tree analysis results

Chi-square Automatic Interaction Detection research revealed that professional experience is the primary predictor of knowledge sufficiency, with the initial division appearing at 6 years of experience. In experience categories, marital status appeared as a secondary predictor, with married persons exhibiting superior knowledge adequacy scores across most experience levels. The professional title appeared as a tertiary predictor in specific specialties, with physicians exhibiting enhanced knowledge adequacy evaluations predominantly among individuals with intermediate levels of professional experience. The analysis revealed subgroups of healthcare professionals at heightened risk for insufficient bisphenol A knowledge: single professionals with 0–5 years of experience (highest risk), married professionals with 0–5 years of experience or single professionals with 6–10 years of experience (medium risk), and married professionals with over 6 years of experience, especially physicians (lower risk).

## 4 Discussion

### 4.1 Primary findings and clinical significance

This comprehensive survey reveals that Turkish healthcare professionals exhibit a significant and pervasive lack of understanding on bisphenol, with 82.6% of participants indicating insufficient knowledge about these prevalent environmental pollutants. This is a significant deficiency in knowledge identified in the study on healthcare workers’ environmental health literacy. It significantly impacts patient safety, workplace health safeguards, and the effectiveness of public health education. Our findings align with and expand upon previous global research. Rouillon et al. (2017) reported that 54.3% of French healthcare professionals were unaware of endocrine-disrupting medicines, and 68.8% lacked knowledge regarding their health impacts (14). Our poll focused just on bisphenols; yet, the revelation that 76.3% of participants were unaware of what bisphenols are indicates that the populace in Türkiye may possess less knowledge about them compared to those in Western European healthcare systems.

The deficiencies in knowledge that were identified have an immediate impact on patient care. Healthcare professionals who lack sufficient knowledge regarding bisphenol are unlikely to be able to offer patients sound advice on how to minimize their exposure, identify situations in which they are at a high risk of exposure, or identify potential health issues associated with bisphenol in clinical settings. Bisphenols were unfamiliar to only 23.7% of healthcare professionals. This demonstrates that there is a fundamental lack of awareness regarding these chemicals as health-relevant environmental variables, rather than merely specific knowledge deficits. The findings of Kumar et al. are consistent with this lack of baseline understanding: dental surgeons in Southern India possessed inadequate knowledge regarding BPA, despite their involvement with products that contained it (15).

The lack of comprehension is particularly concerning due to the numerous sources of BPA in healthcare environments, including medical devices, laboratory apparatus, and patient care items. Li et al. discovered that healthcare personnel had elevated levels of BPA in their urine, indicating that the hazards of occupational exposure are significant and potentially greater than those in other professional settings (25). The healthcare professionals’ lack of knowledge regarding these sources of exposure poses a significant danger to their safety while on the job.

### 4.2 Demographic predictors of knowledge performance

The discovery that professional experience is the most reliable predictor of bisphenol knowledge (rho = 0.144, *p* = 0.004) provides critical insights into the process by which healthcare professionals acquire new knowledge. Healthcare personnel with 6 to 10 years of experience possessed significantly more knowledge than those with 0 to 5 years of experience (4.29 ± 3.48 vs. 3.30 ± 3.15, *p* = 0.049). This finding implies that healthcare workers are not adequately prepared for environmental health challenges by their initial professional education programs, as knowledge development is predominantly achieved through accrued professional experience and continuing education opportunities. Marguillier et al. discovered comparable outcomes when they examined French perinatal health professionals. They stated that environmental health competency necessitates enhanced training throughout the course of professional development (16). The plateau effect observed in knowledge scores of professionals with over a decade of experience suggests that the current methods of continuing education may not be adequately accommodating the requirements of professionals who require additional education in environmental health.

Physicians demonstrated a significantly greater comprehension of bisphenol than nurses (4.64 ± 3.61 vs. 3.29 ± 3.54, *p* = 0.015). Nevertheless, only 21.4% of physicians possessed sufficient expertise, indicating that healthcare professionals of all backgrounds have knowledge deficits. The physicians’ superior performance is likely attributable to their increased exposure to pathophysiology, pharmacology, and toxicology during their medical school years. Nurses, midwives, and health technicians are particularly concerned about their decreased knowledge ratings, as they spend a significant amount of time with patients, which is an ideal opportunity for health education and risk counseling. Similar trends were identified in Asian healthcare systems by Zhang et al. They emphasized the necessity of nurses receiving specialized training in environmental health due to their significant role in coordinating patient care and educating patients (17).

The unexpected but potentially critical discovery is the robust correlation between marital status and bisphenol knowledge (4.26 ± 3.57 for married individuals vs. 3.39 ± 3.30 for single participants, *p* = 0.014). The impact of marital status on health may be attributed to a greater awareness of health issues that is associated with having a family, increased access to health information through family social networks, a stronger desire to learn about environmental health due to concerns about protecting children, or a connection to age and maturity factors that aid in learning and memory capacity.

### 4.3 Occupational health and safety implications

Healthcare professionals are at an elevated risk of exposure to bisphenol due to their involvement in the handling of medical devices, laboratory apparatus, and patient care materials. The following are all prevalent sources of BPA in healthcare settings: intravenous tubing and connections, medical device housings, laboratory equipment and containers, dentistry materials and instruments, incubators and warming devices, and food service materials. Healthcare professionals are incapable of taking the appropriate precautions to safeguard themselves due to their inadequate comprehension of these sources of exposure.

Healthcare personnel may lack sufficient knowledge regarding bisphenol to prevent patients from being exposed to it when they select and employ medical equipment and materials. Sirasanagandla et al. emphasized the necessity for healthcare professionals to be informed about the risks associated with prenatal BPA exposure in order to protect vulnerable populations, particularly due to the potential impact of the chemical on cardiac development (7). Extra protection from bisphenol exposure is necessary for certain patient categories, including expectant women and developing fetuses, preterm infants and neonates, patients with endocrine problems, individuals with cardiovascular illness, and patients who require extensive medical device assistance.

### 4.4 Educational intervention requirements

The identified knowledge gaps indicate that healthcare professional curricula must be revised to incorporate environmental health competencies. Brown et al. examined the comprehension of environmental health among Spanish nurses and emphasized the necessity of incorporating environmental health into the curriculum to enhance competence in this area (18). Educational interventions must encompass fundamental skills such as identifying health risks, safeguarding vulnerable populations, effectively communicating risks, and ensuring workplace safety.

Professional experience serves as a significant predictor of knowledge, indicating that continuing education programs ought to be tailored for individuals at various career stages and in diverse roles. Novice professionals must acquire foundational knowledge in environmental health, whereas experienced individuals should remain informed about recent research and evolving risk assessment methodologies. The variation in knowledge among various professional groups indicates that interprofessional education programs could enhance knowledge consistency and promote collaboration on environmental health issues.

Successful educational interventions require institutional commitment. This entails integrating environmental health topics into mandatory continuing education, developing clinical practice guidelines for environmental health, offering educational resources and decision support tools at the point of care, and establishing consultation services for complex cases. Contemporary educational technologies facilitate the dissemination of knowledge regarding environmental health through various means, including online learning modules featuring interactive case studies, mobile applications providing information at the point of care, virtual reality simulations for training in exposure assessment, and social media platforms for knowledge sharing and updates.

### 4.5 Health policy implications

The lack of knowledge among healthcare professionals regarding certain topics indicates that national health policies should prioritize education on environmental health and enhance professional competency. Rai et al. identified similar deficiencies in the knowledge of occupational health among Turkish healthcare professionals, indicating the necessity for systematic policy reforms (20). Healthcare professionals’ licensing and continuing education requirements ought to incorporate competencies in environmental health. This ensures that all healthcare professionals remain informed about the risks and protective strategies associated with environmental health.

Healthcare facilities must establish comprehensive environmental health policies that address staff training and education, assessment of environmental health risks, patient education and counseling, monitoring and protection of workers from exposure, and procurement of products that are safer than those containing BPA. Environmental health education for healthcare professionals must be integrated into broader public health initiatives to enable healthcare personnel to serve effectively as community environmental health educators and advocates.

## 5 Limitations and future research directions

This study was conducted at a single healthcare facility in southeastern Türkiye, potentially limiting its applicability to other healthcare settings, regions, or countries with varying educational systems and environmental health regulations. The bisphenol knowledge evaluation questionnaire was reviewed by experts; however, it did not undergo comprehensive psychometric testing, such as factor analysis and reliability assessment. The cross-sectional research design captures knowledge levels at a single point in time, limiting its ability to assess changes in knowledge over time or the effectiveness of educational interventions.

Future studies should employ longitudinal designs to examine the temporal changes in knowledge and the effectiveness of educational interventions in enhancing healthcare professionals’ competencies in environmental health. Conducting this examination in various healthcare settings would enhance its generalizability and identify system-specific factors influencing individuals’ comprehension of environmental health. Randomized controlled trials examining targeted educational interventions are essential for developing evidence-based strategies to address identified knowledge gaps. Research examining the impact of healthcare professionals’ insufficient understanding of environmental health on patient outcomes would be beneficial.

## 6 Conclusion

This comprehensive cross-sectional study provides an in-depth examination of the knowledge of bisphenol among Turkish healthcare professionals. It reveals significant deficiencies that adversely impact patient safety, occupational health protection, and the efficacy of public health education. The findings indicate a significant deficiency in knowledge regarding bisphenol among healthcare professionals, with 82.6% demonstrating inadequate understanding and only 23.7% reporting prior awareness of these prevalent environmental toxins. This deficiency in knowledge is evident across all occupational categories and levels of experience.

The identification of professional experience, marital status, and professional title as significant determinants of knowledge performance provides valuable insights for the development of targeted educational interventions. The consistently low levels of knowledge across all demographic groups indicate a necessity for comprehensive systemic educational reforms, rather than solely targeted interventions.

The results indicate the necessity of incorporating environmental health education into the training and continuing education programs for healthcare professionals. Healthcare professionals are unable to adequately assess environmental health risks, advise patients on exposure reduction, implement appropriate occupational safety measures, or serve as reliable sources of environmental health information for their communities due to established knowledge gaps.

The study’s conclusions directly influence the improvement of healthcare policy, the modification of educational programs, and the enhancement of clinical practice. Healthcare organizations must prioritize the enhancement of environmental health knowledge through diverse teaching methods, policy modifications, and comprehensive system adjustments that address both individual knowledge deficits and structural barriers to effective environmental health practice.

Future research should concentrate on developing and evaluating evidence-based educational interventions, expanding assessments to encompass a broader spectrum of healthcare settings and populations, and investigating the impact of healthcare professionals’ comprehension of environmental health on patient outcomes. Healthcare systems must implement a comprehensive range of efforts to adequately prepare personnel for addressing the environmental health challenges of the 21st century, ensuring the safety of both patients and healthcare workers.

## Data Availability

The original contributions presented in the study are included in the article/supplementary material, further inquiries can be directed to the corresponding author.
